# Correction to “Atypical Guillain‐Barré Syndrome Preceding Icteric Hepatitis A: A Diagnostic Challenge—A Case Report”

**DOI:** 10.1002/ccr3.72612

**Published:** 2026-04-23

A. Afana, A. H. Sharaf, J. Omar, A. Elkhazin, H. Salameh, “Atypical Guillain‐Barré Syndrome Preceding Icteric Hepatitis A: A Diagnostic Challenge—A Case Report,” *Clinical Case Reports* 14 no. 4 (2026):e72364. https://doi.org/10.1002/ccr3.72364.

There has been an error in the published affiliations of two authors.

The accurate affiliations should be as follows:

• Ahmed H. Sharaf: Faculty of Medicine, Kafr Elsheikh University, Kafr Elsheikh, Egypt.

• Ahmad Elkhazin: Faculty of Medicine, Alexandria University, Alexandria, Egypt.

The current publication incorrectly lists both authors under the same affiliation.

We apologize for this error.

